# RNA interference of a trehalose‐6‐phosphate synthase gene reveals its roles in the biosynthesis of chitin and lipids in *Heortia vitessoides* (Lepidoptera: Crambidae)

**DOI:** 10.1111/1744-7917.12650

**Published:** 2018-12-11

**Authors:** Jing‐Xiang Chen, Zi‐Hao Lyu, Chun‐Yan Wang, Jie Cheng, Tong Lin

**Affiliations:** ^1^ College of Forestry and Landscape Architecture South China Agricultural University Guangzhou China

**Keywords:** chitin biosynthesis, *Heortia vitessoides* Moore, lipid biosynthesis, metamorphosis, RNA interference, trehalose‐6‐phosphate synthase

## Abstract

Trehalose‐6‐phosphate synthase (TPS), an enzyme that hydrolyzes two glucose molecules to yield trehalose, plays a pivotal role in various physiological processes. In this study, we cloned the trehalose‐6‐phosphate synthase gene (*HvTPS)* and investigated its expression patterns in various tissues and developmental stages in *Heortia vitessoides* Moore (Lepidoptera: Crambidae). *HvTPS* was highly expressed in the fat body and after pupation or before molting. We knocked down *TPS* in *H. vitessoides* by RNA interference and found that 3.0 *μ*g of ds*HvTPS* resulted in optimal interference at 24 h and 36 h post‐injection and caused a sharp decline in the survival rate during the 5th instar larval–pupal stage and obviously abnormal or lethal phenotypes. Additionally, compared to the controls, TPS activity and trehalose contents were significantly lower and the glucose content was significantly higher 24 h or 36 h after injection with 3.0 μg of ds*HvTPS*. Furthermore, the silencing of *HvTPS* suppressed the expression of six key genes in the chitin biosynthesis pathway and one key gene related to lipid catabolism. The expression levels of two genes associated with lipid biosynthesis were upregulated. These results strongly suggest that *HvTPS* is essential for the normal growth and development of *H. vitessoides* and provide a reference for further studies of the utility of key genes involved in chitin and lipid biosynthesis for controlling insect development.

## Introduction

*Heortia vitessoides* Moore (Lepidoptera: Crambidae) is regarded as the most serious pest affecting the normal growth of *Aquilaria sinensis*, which is an endangered tree species (Liu *et al*., 2013). They consume all leaves of *A. sinensis* in a few days, causing severe economic losses (Qiao *et al*., 2012). To date, studies of *H. vitessoides* have mainly focused on ecological and biological properties, and studies of other aspects, such as molecular and physiological characteristics, are limited. Therefore, more extensive and comprehensive studies of *H. vitessoides* are necessary.

Trehalose is a highly stable non‐reducing disaccharide consisting of two glucose molecules linked by an *α*,*α*‐1,1‐glycosidic bond (Birch, 1962; Chen & Haddad, 2004). Trehalose is present in a wide variety of organisms; it has been found in over 80 species, including plants, algae, fungi, yeasts, bacteria, insects and other invertebrates (Elbein, 1974; Becker *et al*., 1996). In the animal kingdom, trehalose was first reported in insects (Elbein *et al*., 2003). Trehalose accounts for 80%–90% of total sugar in the hemolymph of insects; thus, it is referred to as hemolymph sugar in insects (Becker *et al*., 1996; Elbein *et al*., 2003). Recent studies of trehalose have revealed multiple physiological roles in insects, not only as a storage molecule, but also for protection against abiotic stresses, such as high or low temperatures, nutrition or starvation, oxidation, high osmotic pressure, toxic substances, and UV‐B irradiation (Ribeiro *et al*., 1997; Rangel *et al*., 2008; Feofilova *et al*., 2014; Tamang *et al*., 2016). These findings indicate that trehalose is pivotal for the growth, development and survival of insects (Xu *et al*., 2009).

At least five biosynthetic pathways of trehalose have been described (Avonce *et al*., 2006). In most species, including insects, biosynthesis occurs ubiquitously by two enzymatic steps catalyzed by trehalose‐6‐phosphate synthase (TPS) and trehalose‐phosphatase (TPP) (Elbein *et al*., 2003). *TPS* in insects is a fused gene that encodes two conserved functional domains in tandem: *TPS*, a homolog of Ost A of *Escherichia coli*, and *TPP*, a homolog of Ost B of *E. coli* (Su *et al*., 1994; Chung, 2008). The insect *TPS* gene was first identified and cloned from *Drosophila melanogaster* in 2002 (Chen *et al*., 2002). To date, numerous *TPS* genes have been cloned in various insects, such as *Locusta migratoria manilensis* (Cai & Xia, 2009), *Spodoptera exigua* (Tang *et al*., 2010), *Nilaparvata lugens* (Yang *et al*., 2017) and *Catantops pinguis* (Tang *et al*., 2011). However, it has not been identified in *Heortia vitessoides* and its detailed role in insect physiology has not been well addressed

In insects, functional studies of *TPS* are mainly focused on the effects of chitin biosynthesis. Chitin is the main component of the insect cuticle and interacts with cuticular proteins to form a complex structure that could stabilize the cuticle, while maintaining skin elasticity and other physical properties (Moussian, 2010). In insects, the chitin biosynthesis pathway, which was first described by Candy and Kilby (1962), is a highly complex physiological and biochemical process. Trehalose is mainly synthesized via *TPS* and has been suggested as an initial substrate for chitin synthesis (Merzendorfer & Zimoch, 2003). Transforming trehalose into chitin could be successfully accomplished by a pathway including various enzymes (Chen *et al*., 2010). Most studies of this pathway have focused on trehalase and chitin synthase. The effects of *TPS* on chitin synthesis pathways in *H. vitessoides* are not clear. However, few studies have linked the *TPS* gene to the synthesis of chemicals other than chitin. According to previous studies, trehalose is converted to glucose, which is involved in the biosynthesis of macromolecular organic compounds, for example chitin biosynthesis (Shen *et al*., 2017), lipid biosynthesis (Shi *et al*., 2016b) and carbon metabolism (Avonce *et al*., 2006). Studies have shown that double‐stranded *TPS* (ds*TPS*)‐fed larvae consume more leaves than controls when measured on day 3 after the initiation of the bioassay (Shi *et al*., 2016b). In addition, we found that ds*HvTPS* injection did not significantly affect larval feeding in this study (data not shown). As a result, we hypothesized that insects absorb an equivalent amount of sugar or more from food, and the mechanism by which glucose is converted to trehalose is blocked by knocking down the *TPS* gene, which is predicted to increase the activity of other synthetic pathways because the reduced glucose content can facilitate carbohydrate absorption in insects (Chapman, 1998). In addition to the glycogen conversion pathways, are there other mechanisms by which the glucose content is reduced in insects? In other words, can hemolymph glucose be converted to other chemicals when the biosynthesis of trehalose is inhibited by silencing *HvTPS*?

In this study, we cloned and characterized a *TPS* gene from the holometabolous Lepidoptera species *Heortia vitessoides*. Reverse transcription quantitative polymerase chain reaction (RT‐qPCR) was used to analyze the expression patterns of *HvTPS* at various developmental stages and in various tissues. Furthermore, we used an *in vivo* RNA interference (RNAi) method to efficiently disrupt *HvTPS* gene function in order to elucidate its role in the development of *H. vitessoides*, including phenotypic observations, development time, mortality, larval chitin and lipid biosynthesis. The results will contribute to a better understanding of the role of *HvTPS* in metamorphosis and the chitin and lipid biosynthesis pathways.

## Materials and methods

### Insect rearing and sampling

*H. vitessoides* larvae were reared on the leaves of *A. sinensis* at a controlled temperature (25°C) and relative humidity (70% ± 5%), and a photoperiod of 14 : 10 L : D. To investigate the stage‐specific expression profiles of the target gene, 4–5th instar larvae, early (1‐day‐old pupae) and late pupae (15‐day‐old pupae), 5‐day‐old and 10‐day‐old pupae, and 1‐day‐old and 2‐day‐old adults were used for experiments. To examine the tissue‐specific expression profiles of the target gene, 5th instar insects were dissected to collect separate tissues (head, midgut, fat body, Malpighian tubules and epidermis tissues). These samples were dissected in phosphate‐buffered saline (PBS), snap‐frozen in liquid nitrogen, and stored at –80 °C until further use.

### Molecular cloning

Based on the transcriptome database of *H. vitessoides* (Cheng *et al*., 2017), a putative fragment of *HvTPS* with the 5′ end of the open reading frame (ORF) was selected. For the whole ORF, two specific primers were designed for the amplification of the 3′ end using Primer 5.0 (all primer sequences are presented in supplementary data, Table S1). Using SMART rapid amplification of complementary DNA ends PCR and nested PCR, 3′ end cloning was performed. The first PCR was performed using the general primers UMP and the specific primer *TPS*‐F1. The PCR conditions were as follows: predenaturation at 94 °C for 5 min, degeneration at 94 °C for 40 s, annealing at 60 °C for 1 min, extension at 72 °C for 2 min for 30 cycles, followed by extension at 72 °C for 10 min. Then, 0.1 *μ*L of PCR product was used as a template for the second PCR, which was performed using the common primers NUP and specific *TPS*‐1F2, following the same methods used for the first PCR. The PCR product was recovered and purified. The purified DNA was ligated into the pClone007 Vector (Tsingke, Beijing, China) and sequenced using the dideoxynucleotide method.

### Sequence analysis and phylogenetic tree construction

The amino acid sequence encoded by *HvTPS* was compared to the TPS sequences of other insects, which were retrieved from GenBank databases (http://www.ncbi.nlm.nih.gov/). The protein molecular mass (kDa) and isoelectric point (pI) were calculated based on the amino acid sequence using the ExPASy online Server (http://cn.expasy.org/tools/pi_tool.html). Sequence comparisons were performed using DNAMAN. Phylogenetic tree construction was performed using MEGA 4.0 and ClustalX 1.83 with the neighbor‐joining method and support was determined based on 1000 bootstrap replicates.

### Preparation of dsRNA and injection

dsRNA fragments targeting *HvTPS* (432 bp) and *GFP* (400 bp) were prepared using the T7 RiboMAX™ Express RNAi System Kit (Promega, Madison, WI, USA). In brief, primers containing T7 RNA polymerase promoter sequences were designed for PCR to obtain DNA templates (all primer sequences are presented in supplementary data, Table S1). The DNA templates for *HvTPS* and *GFP* (control) were subsequently used in a transcription reaction with the T7 RNA polymerase to generate dsRNA fragments. The DNA template was removed, followed by dsRNA annealing and removing ssRNA (single‐stranded RNA) under nuclease digestion. Then, the dsRNA was purified according to the purification protocol of the manufacturer (Promega). Finally, the concentration of dsRNA was detected using a NanoDrop 2000 spectrophotometer (Thermo Fisher Scientific, Waltham, MA, USA). The integrity and purity of dsRNA fragments were assessed by 1.5% agarose gel electrophoresis. Samples were then stored in a refrigerator at –80 °C until use.

Ds*HvTPS* doses of 5, 4, 3, 2 and 1 *μ*g were used to determine RNAi sensitivity in 4th instar larvae of the same size. The treatment method, including the microinjection of dsRNA, generally followed that described by Xiong *et al*. (2016). Briefly, 1 *μ*L of purified ds*HvTPS* (5 *μ*g/*μ*L, 4 *μ*g/*μ*L, 3 *μ*g/*μ*L, 2 *μ*g/*μ*L, and 1 *μ*g/*μ*L) was injected into the antepenultimate abdominal segments of each 4th instar larvae respectively using FemtoJet (Eppendorf, Hamburg, Germany). There were three controls for all ds*HvTPS* treatments, that is, diethyl pyrocarbonate water (1 *μ*L), ds*GFP* (equivalent volume) and blank controls. Larvae of *H. vitessoides* were administered via leaves of *A. sinensis*. There were 30 larvae in each treatment group, and experiments were repeated independently three times.

### Quantitative real‐time PCR

Primer 5.0 was used to design quantitative real‐time PCR primers. All primer sequences are displayed as supplementary data in Table S1. The relative expression levels of target genes were measured by quantitative real‐time PCR and were normalized to the stable reference gene *α*‐tubulin (GenBank accession: MG132200) (Cheng *et al*., 2018). Each reaction system contained 10.0 *μ*L of SYBR Premix (Takara, Shiga, Japan), 7.2 *μ*L of ddH_2_O, 0.4 *μ*L of forward and reverse primers (10 *μ*mol/L), and 2.0 *μ*L of cDNA. Quantitative real‐time PCR was performed using SYBR Supermix (Bio‐Rad, Hercules, CA, USA) according to the manufacturer's protocol. The amplification program was as follows: 95 °C for 5 min, 95 °C for 10 s, 60 °C for 20 s; 40 cycles from steps 2 to 3. PCR was performed in a 96‐well plate, and the results were analyzed using the LightCycler® Real Time PCR System (Roche Diagnostics, Indianapolis, IN, USA). Three biological replicates and three technical replicates were set for quantitative real‐time PCR analysis. The 2^−∆∆^ ${}^{C{}_{t}}$ method was used to assess the variation in quantitative transcript levels (Livak & Schmittgen, 2001).

### Measurement of TPS activity after RNAi

TPS activity in crude extracts of insects subjected to different treatments were measured according to the Hottiger method (Hottiger *et al*., 1987). The products, trehalose‐6‐phosphate, were generated during the reaction, and were quantitatively determined utilizing the Anthrone method. One unit of TPS activity was defined as the amount of TPS producing 1.0 *μ*mol/L 6‐phosphate‐trehalose per minute under assay conditions. The protein content for *H. vitessoides* after each treatment was determined using Easy Protein Quantitative Kit (Bradford) (TransGen, Beijing, China). The standard protein was bovine serum albumin. The activity of HvTPS was expressed as *μ*mol UDP/mg protein/min.

### Analysis of trehalose and glucose contents

Trehalose and glucose contents were measured using a Trehalose and Glucose Assay Kit (Comin, Jiangsu, China) according to the manufacturer's protocol. Briefly, 24 h and 36 h after injection with 3.0 *μ*g of ds*HvTPS*, 0.1 g of the sample was added to 1 mL of extraction solution, PBS (pH 5.8), and homogenized in an ice bath. After sitting at room temperature for 45 min, they were oscillated 3–5 times and allowed to cool. Samples were centrifuged at 25 °C, 8000× *g* for 10 min, and the supernatant was obtained. Then, 60 *μ*L of the supernatant and 240 *μ*L of working solution were thoroughly mixed in an EP tube. After incubation in a 95 °C water bath for 10 min, it was cooled to room temperature. Then, 200 *μ*L of the mixed solution in a Quartz Microplate was used to obtain the absorbance at 620 nm, which was utilized to calculate the trehalose content. For the glucose content assay, 0.1 g of sample was homogenized in 1 mL of ddH_2_O and incubated in a 95 °C water bath for 10 min (covered tightly to prevent moisture loss), cooled on ice, and centrifuged at 25 °C, 8000× *g* for 10 min to obtain the supernatant. Then, 20 *μ*L of supernatant and 180 *μ*L of reaction reagent were thoroughly mixed in an EP tube, and incubated at 25 °C for 15 min. The concentration of glucose in the supernatant was calculated by measuring absorbance at 505 nm.

### Chitin analysis

Five individuals were randomly chosen from larvae injected with 3 *μ*g of ds*HvTPS* at 12 h or 72 h and from the control groups (ds*GFP*). They were utilized to detect the contents of the epidermis and midguts of *H. vitessoides* larvae according to the method described by Arakane *et al*. (2005). Three independent biological replicates were used for assays.

### Fat body analysis

Five larvae were randomly chosen from larvae injected with 3 *μ*g of ds*HvTPS* at 72 h and from the control groups (ds*GFP*). After the larvae were dissected under a stereoscope (Leica M165C, Wetzlar, Germany), fat bodies were collected and images were obtained. Finally, the collected fat bodies were put into a centrifuge tube and measured using a Micro‐electronic balance (Mettler Toledo AB104‐S, Greifensee, Switzerland). Three independent biological replicates were used for assays.

### Statistical analysis

Data were analyzed using SPSS 16.0 (SPSS Inc., Chicago, IL, USA, 2008) and results are presented as means ± SE (standard error). Significant differences between treatments were analyzed using Student's *t*‐tests (for comparisons of two means) or by one‐way analysis of variance (ANOVA) followed by a least significant difference (LSD) test (for multiple comparisons).

## Results

### Sequence analysis of HvTPS cDNA

**Figure 1:**
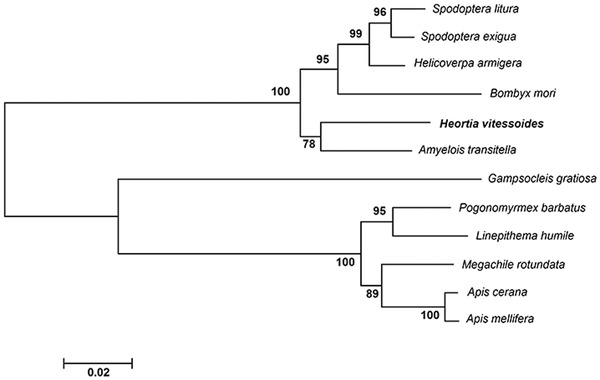
Phylogenetic tree of trehalose phosphate synthases (TPS) in *Heortia vitessoides* and other insects. The tree was constructed by the neighbor‐joining method using MEGA. The sequences were obtained from GenBank under the following accession numbers: *Spodoptera litura* (ADA63844), *Spodoptera exigua* (ABM66814), *Helicoverpa armigera* (XP_021201246), *Bombyx mori* (XP_004926812), *Amyelois transitella* (XP_013187220), *Gampsocleis gratiosa* (APZ77037), *Pogonomyrmex barbatus* (XP_011641245), *Linepithema humile* (XP_012234592), *Megachile rotundata* (XP_003702415), *Apis cerana* (XP_016905400) and *Apis mellifera* (XP_00324923). Scale bar represents 0.02 substitutions per site. Numbers at nodes indicate bootstrap support.

Based on *H. vitessoides* transcriptome data, cloning and identification of the whole ORF of *HvTPS* (GenBank accession number: MG787167) was performed. *HvTPS* cDNA contained an ORF of 2496 nucleotides, encoding a protein of 831 amino acids with a predicted mass of approximately 93.48 kDa and a pI of 7.06. A BLAST analysis and multiple sequence alignment of TPSs from different insects revealed that HvTPS shares 70%–96% identity with other known TPSs; additionally, there were two putative catalytic domains and two signature motifs (HDYHL and DGMNLV) (Fig. S1). A phylogenetic analysis showed that the TPSs of *H. vitessoides* and *Amyelois transitella* were similar and were clearly distinct from the TPSs of other insects (Fig. 1).

### Tissue‐specific and developmental stage‐specific expression patterns of HvTPS

**Figure 2:**
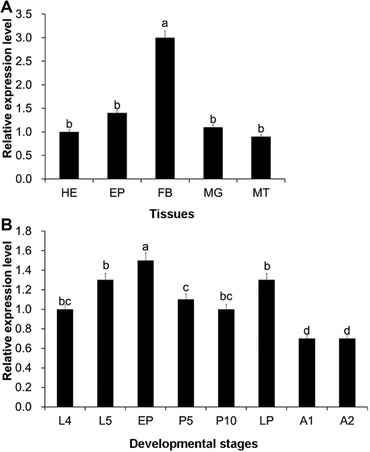
Relative expression levels of *HvTPS* in different tissues (A) and at different developmental stages (B) of *Heortia vitessoides*. Head (HE), epidermis (EP), fat body (FB), midgut (MG) and Malpighian tubules (MT). L4–L5, 4th and 5th instar larvae; EP, early pupae; P5 and P10, 5‐day‐old and 10‐day‐old pupae; LP, late pupae; A1–A2, 1‐day‐old and 2‐day‐old adults. Error bars represent the standard error of the calculated means based on three biological replicates. Different letters above the error bars indicate significant differences (*P* < 0.05).

Quantitative real‐time PCR experiments were performed to determine the tissue‐ and stage‐specific expression patterns of *HvTPS*. In a tissue expression analysis, *HvTPS* expression was detected in all tissues examined in this study, and fat body expression was the highest among these tissues (Fig. 2A). With respect to developmental stage, *HvTPS* was expressed at all tested stages, but was highly expressed after pupation or before molting in *H. vitessoides* (Fig. 2B).

### Efficiency of RNAi

**Figure 3:**
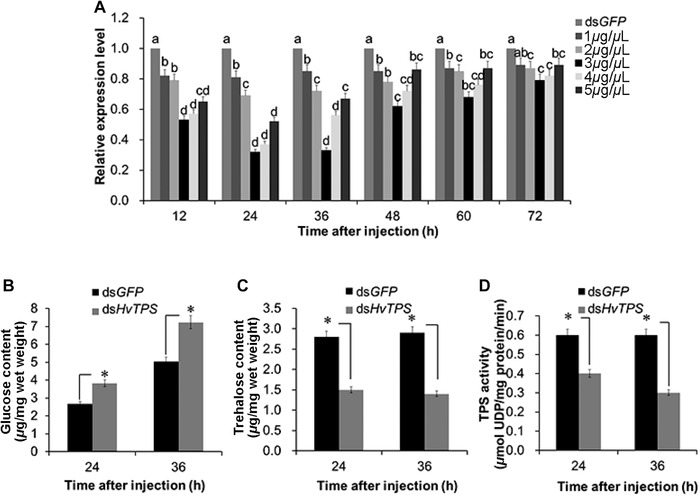
Changes in the messenger RNA (mRNA) and enzyme activity levels of *HvTPS* and the glucose or trehalose content after specific RNA interference (RNAi). (A) Relative expression levels of *HvTPS* in 4th‐instar larvae after injection with ds*HvTPS* at doses of 1.0, 2.0, 3.0, 4.0 and 5.0 *μ*g/individual. (B) Changes in the glucose content after RNAi. (C) Changes in the trehalose content after RNAi. (D) Changes in HvTPS activity after ds*HvTPS* injection. Error bars represent the standard error of the calculated means based on three biological replicates. Different letters above the error bars indicate significant differences among treatments and the control measured at the same time (*P* < 0.05).

To determine the optimal dose of ds*HvTPS* for efficient RNAi, a series of ds*HvTPS* concentrations (1, 2, 3, 4 and 5 *μ*g/*μ*L) were tested by injection into the 3rd abdominal segment of 4th instar larvae of *H. vitessoides* with a volume of 1 *μ*L. Quantitative real‐time PCR was used to detect the expression of *HvTPS* after treatment with different doses of ds*HvTPS*. The mRNA transcript levels of *HvTPS* were lower at 12, 24, 36, 48, 60 and 72 h after injection than those in the control groups (ds*GFP*). In particular, a concentration of 3.0 *μ*g/*μ*L ds*HvTPS* led to a higher RNAi efficiency at 24 h and 36 h post‐injection. *HvTPS* transcript levels recovered to some extent 48 h after injection but were still lower than those in controls (Fig. 3A).

Furthermore, compared to those of the control, TPS activity and trehalose contents were significantly lower, but the glucose content was significantly higher 24 h or 36 h after injection with 3.0 *μ*g of ds*HvTPS* (Fig. 3B, 3C, and 3D), demonstrating the success of RNAi and suggesting that the down‐regulation of *HvTPS* has a crucial impact on the trehalose and glucose contents in *H. vitessoide*s.

### Survival rates and phenotype analysis after injection with dsRNA

**Figure 4:**
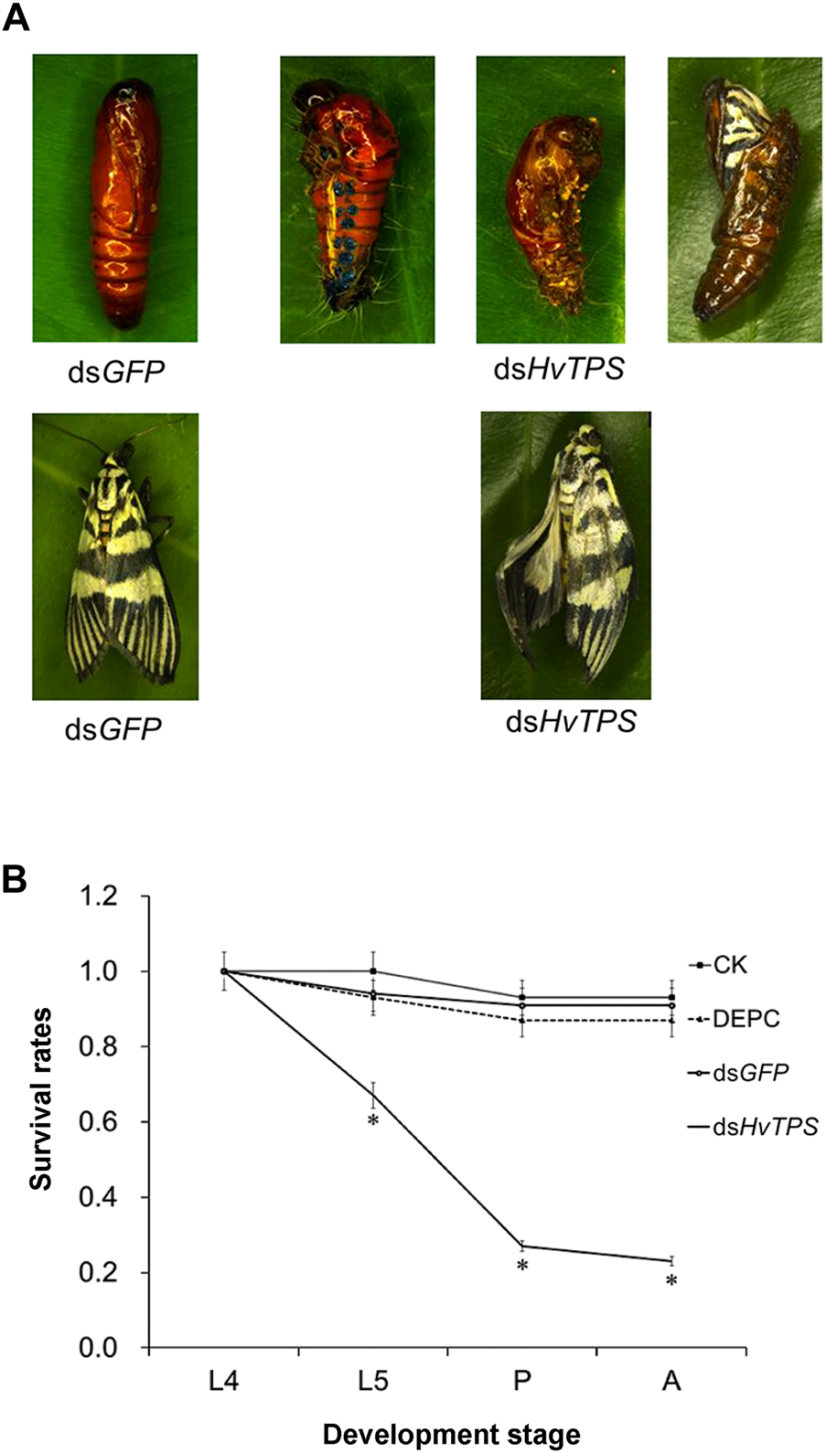
Analysis of phenotypes and survival rates after injection with ds*HvTPS*. (A) During the larval–pupal stage, insects produced three lethal phenotypes: severe‐abnormal, abdomen‐abnormal and half‐eclosion. During the pupal‐adult stage, insects produced one lethal phenotype: misshapen wings. (B) Survival rates after the injection of ds*HvTPS*. The survival rates of insects after ds*HvTPS* injections for 4th instar larvae to adults. Error bars represent the standard error of the calculated means based on three biological replicates. Different letters above the error bars indicate significant differences between treatments and controls measured at the same time point (*P* < 0.05).

The survival rates of the insects injected with 3.0 *μ*g of ds*HvTPS* in 4th instar larvae during development were 67% (5th instar larvae), 27% (pupae) and 23% (adults) post‐injection, which were significantly lower than those of the insects in the three control groups. A sharp decline in the survival rate was observed between 5th instar larvae and pupae after injection with ds*HvTPS* (Fig. 4B). Furthermore, a phenotype analysis also showed that during larval‐pupal metamorphosis, individuals injected with 3 *μ*g of ds*HvTPS* had obviously abnormal or lethal phenotypes (Fig. 4A). In total, 40% of all individuals could not successfully pupate and died, partially wrapped in the larval cuticle, with deformed larvae or pupae. However, 23% of individuals became adults, but with a delay. Among these individuals, the proportion with “misshapen wings” was 15%. No obvious change in phenotype was observed in the ds*GFP* control.

### Chitin in HvTPS RNAi larvae

**Figure 5:**
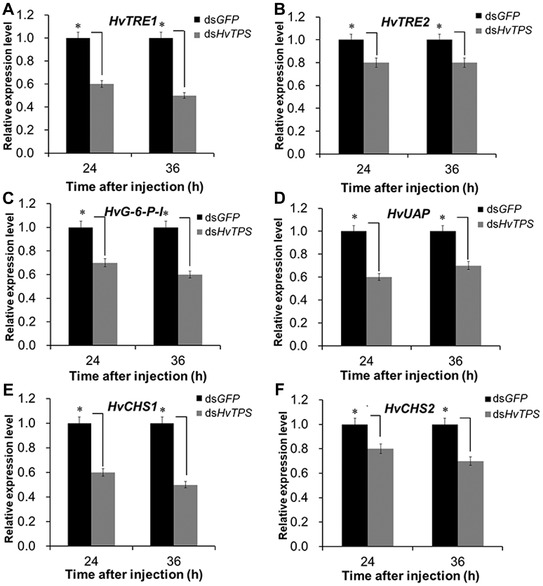
Relative expression levels of key genes in the chitin biosynthesis pathway after RNA interference (RNAi). The relative expression levels of *HvTRE‐1* (A), *HvTRE‐2* (B), *HvG6PI* (C), *HvUAP* (D), *HvCHS‐1* (E), and *HvCHS‐2* (F) in *Heortia vitessoides* at 24 h and 36 h after injection with 3.0 *μ*g of ds*HvTPS*. Error bars represent the standard error of the calculated means based on three biological replicates. Different letters on the error bars indicate significant differences (*P* < 0.05).

After the injection of 3 *μ*g of ds*HvTPS* for 12 h in 4th instar larvae, chitin contents of the epidermis (Ep) and midguts (Mg) exhibited little change in *H. vitessoides* larvae compared to those of the control groups (Table 1). However, when measured at 72 h, compared with the control group, the chitin contents in the Ep and Mg of larvae injected with 3 μg of ds*HvTPS* were slightly lower and the average amount of chitin in each larva was reduced by one‐thirteenth. Similarly, the mRNA transcript levels of six genes (Trehalose, *HvTRE1* and *HvTRE2*; glucose‐6‐phosphate isomerase, *HvG‐6‐P‐I*; UDP‐*N*‐acetylglucosamine pyrophosphorylase, *HvUAP*; *chitin synthases*, *HvCHS1*, and *HvCHS2*) associated with chitin biosynthesis were all significantly decreased at 24 h and 36 h after the knockdown of *HvTPS* (Fig. 5A–5F). These results indicate that the knockdown of *HvTPS* inhibits chitin biosynthesis in *H. vitessoides*.

**Table 1:** Effects of the injection of ds*HvTPS* on the chitin content in the epidermis (Ep) and midguts (Mg) of *Heortia vitessoides* larvae

Tissues	Group	Content at 12 h (*μ*g/individual)	Content at 72 h (*μ*g/individual)
Ep	CK	62.13 ± 0.52a	70.26 ± 0.43a
	ds*GFP*	62.23 ± 0.71a	69.78 ± 0.32a
	ds*HvTPS*	61.15 ± 0.43a	64.27 ± 0.11b
Mg	CK	1.91 ± 0.12a	2.02 ± 0.09a
	ds*GFP*	1.88 ± 0.15a	2.02 ± 0.10a
	ds*HvTPS*	1.83 ± 0.10a	1.84 ± 0.07b

Each data point is the mean ± SEM of three independent experiments with ten individuals each (*n* = 30). Different letters indicate significant differences of the chitin content (*P* < 0.05, Least significant ranges, SPSS).

### Lipid content in HvTPS RNAi larvae

**Figure 6:**
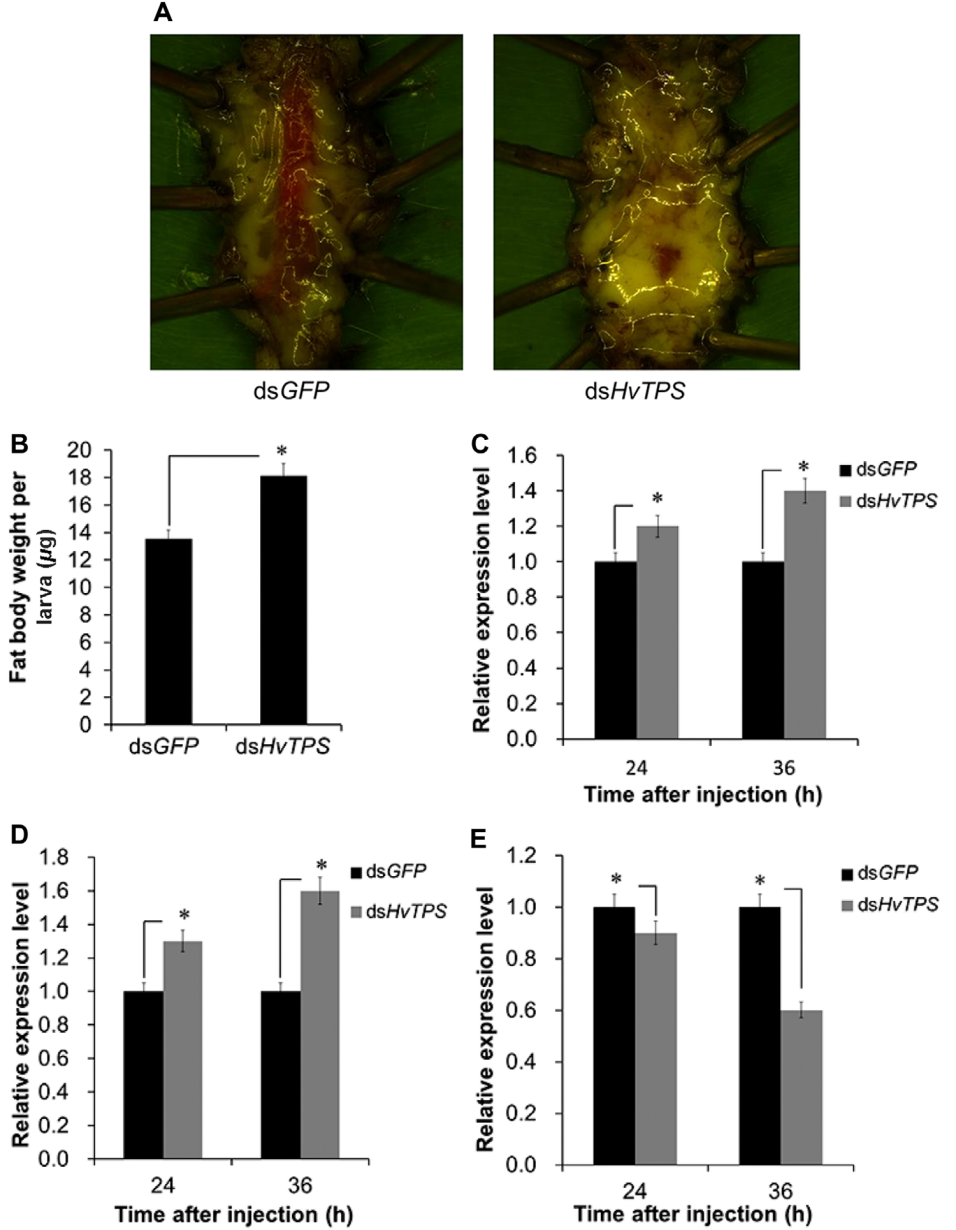
Changes in lipids in *HvTPS* RNA interference (RNAi) larvae. (A) Fat body size and (B) fat body weight after larvae were injected with 3 *μ*g of ds*HvTPS* and dissected at 72 h. Expression levels of two genes related to fatty acid biosynthesis, (C) acetyl‐CoA carboxylase (*HvACC*) and (D) fatty acid synthase (*HvFAS*), and (E) lipase 1 (*HvLIP1*) associated with lipid degradation at 24 h and 36 h after the knockdown of *HvTPS*. Error bars represent the standard error of the calculated means based on three biological replicates. Different letters on the error bars indicate significant differences (*P* < 0.05).

After larvae injected with 3 *μ*g of ds*HvTPS* at 72 h were dissected; the fat body filled the bodies of insects and could substantially cover all internal organs. In contrast, some internal organs in the bodies of insects, such as the tracheae and alimentary canal, were clearly observed in the control group (ds*GFP*) (Fig. 6A). In addition, the fat body weight was higher in the treated group than in the controls (Fig. 6B). In accordance with the above results, the expression levels of two genes related to fatty acid biosynthesis, acetyl‐CoA carboxylase (*HvACC*) and fatty acid synthase (*HvFAS*), increased substantially at 24 h and 36 h after the knockdown of *HvTPS* (Fig. 6C and 6D). In contrast, lipase 1 (*HvLIP1*), which is associated with lipid degradation, was downregulated (Fig. 6E). These results indicate that *HvTPS* was involved in lipid biosynthesis in *H. vitessoides*.

## Discussion

In this study, tissue‐specific expression profiles revealed that *HvTPS* was specifically expressed in the fat body at the larval stage, consistent with the expression of *HaTPS* from *Helicoverpa armigera* (Xu *et al*., 2009), *SeTPS* from *Spodoptera exigua (*Tang *et al*., 2010) and *CpTPS* from *Catantops pinguis* (Tang *et al*., 2011). These results are in accordance with the fact that trehalose is synthesized in the fat body in insects and then released into the hemolymph (Elbein *et al*., 2003). The expression of *HvTPS* was detected in all developmental stages examined in the study, indicating that it might play distinct roles during insect growth and development. These results are similar to those of a previous study (Shi *et al*., 2106a) showing that *LdTPS* is expressed primarily in the larval and pupal stages. The expression levels of *BmTPS* were high during pupation or molting in *Bactrocera minax* (Xiong *et al*., 2016). Consistent with these reports, we also observed high *HvTPS* expression after pupation or before molting, and this was probably related to the requirement for chitin during metamorphosis. In addition, relative expression levels of *HvTPS* were higher during the pupal stage than at other time points. Previous studies have demonstrated a significant increase in the expression of *BmTPS* in *B. minax* during the whole pupal stage (Xiong *et al*., 2016), which may be related to protective mechanisms, for example, protection against abiotic stresses and regulation of pupal diapause in *Delia antiqua* (Guo *et al*., 2015).

Silencing *HvTPS* severely affected the total developmental process, especially metamorphosis at the larval–pupal and pupal–adult stages. It had obviously negative effects, that is, larval or pupal growth was retarded, the periods of larval development and eclosion were delayed, and deformed pupae and adults were observed. Survival rates decreased dramatically during larval‐pupal metamorphosis. In agreement with our results, RNAi of *BmTPS* increased larval mortality and led to lethal or malformed phenotypes in *B. minax* (Xiong *et al*., 2016). The knockdown of *LdTPS* decreased the trehalose content and caused larval and pupal lethality and delayed development (Shi *et al*., 2016b). After RNAi, sufficient glucose was obtained by feeding on large amounts of leaves to maintain the energy supply at the 4th and 5th instar stages, but synthesis of trehalose was blocked and its content decreased. When the larvae stop feeding at the mature larval stage or pupal stage, it resulted in a lack of energy supply and affected chitin synthesis during metamorphosis for the deficiency in the early synthesis of trehalose in the hemolymph. There may be a reason for larval and pupal death. Moreover, insufficient trehalose would reduce protection against abiotic stresses. Consequently, it caused larval and pupal death and survival rates of less than 30% during the pupal stage. It also caused obvious abnormal phenotypes, including abnormal pupae or adults, and the extension of development time. These phenotypes were observed when *HvTPS* gene expression was knocked down, indicating that RNAi is an effective strategy for *H. vitessoides* pest management.

The chitin biosynthesis pathway regulates the main component of the cuticle of most insects and the peritrophic membranes of arthropods. In insects, trehalose, mainly synthesized by *TPS*, is believed to be a main substrate for chitin biosynthesis (Shukla *et al*., 2015). Eight enzymes are involved in the insect chitin biosynthesis pathway, starting with trehalose (Chen *et al*., 2010). In this study, we found that the knockdown of *HvTPS* not only reduced the relative expression level of *HvTPS*, but also decreased the hemolymph trehalose content and TPS activity 24 h or 36 h post‐injection with 3 *μ*g of ds*HvTPS*, demonstrating the success of RNAi. In addition, a dose of 3.0 *μ*g of ds*HvTPS* resulted in high RNAi efficiency 24 h and 36 h post‐injection, suggesting that this was the optimal dose to silence *HvTPS* in *H. vitessoides*. When *TRE* genes were silenced in *S. exigua* (Tang *et al*., 2010), *Tribolium castaneum* (Chen *et al*., 2017) and *Nilaparvata lugens* (Zhang *et al*., 2017), *G‐6‐P‐I*, *UNAP*, *UAP*, *CHSA* (*HvCHS1*) and *CHSB* (*HvCHS2*) gene expression levels decreased, indicating that *TRE* genes affect chitin biosynthesis. These findings are consistent with our results that the expression levels of four key chitin biosynthesis genes (*HvG‐6‐P‐I*, *HvUAP*, *HvCHS1*, and *HvCHS2*) in *HvTPS* RNAi specimens were lower than those in the controls when *H. vitessoides TREs* (*HvTRE1* and *HvTRE2*) were inhibited by silencing *HvTPS*. The results revealed that the chitin biosynthesis pathway can be regulated by *HvTPS* genes at the mRNA level in *H. vitessoides*. Moreover, the chitin content decreased slightly compared with those in controls, as in ds*LdTPS*‐injected larvae of *Leptinotarsa decemlineata* (Shi *et al*., 2016b). After injecting ds*NlCHSA* into *N. lugens*, the chitin content decreased (Li *et al*., 2017), consistent with our results showing *H. vitessoides HvCHS1* and *HvCHS2* gene inhibition after the silencing of *HvTPS*. Accordingly, these findings collectively indicated that *HvTPS* could be an important factor involved in chitin biosynthesis and thus could affect metamorphosis in *H. vitessoides* development.

Insects absorb carbohydrates, mainly in the form of monosaccharides, by passive transport. Reducing the monosaccharide content in the hemolymph may promote the absorption of monosaccharides, such as trehalose, in fat bodies (Chapman, 1998). In addition, there may be other ways to reduce the monosaccharide content in the hemolymph of insects, including glycogen synthesis, glycolysis and pentose phosphate pathways (Garrett & Grisham, 2010). Therefore, we presumed that knocking down *TPS* in *H. vitessoides* would affect trehalose biosynthesis and thus may facilitate other metabolic pathways to lower hemolymph monosaccharides. The end product of glycolysis, pyruvate, could subsequently be used in the citrate cycle, fatty acid biosynthesis, and proline biosynthesis (Garrett & Grisham, 2010). In this study, we focused on fatty acid biosynthesis metabolites and three key genes encoding enzymes involved in fatty acid biosynthesis in the ds*GFP* and ds*HvTPS* group. Our results indicate that lipid accumulation is greater in the *HvTPS* RNAi larvae, thereby decreasing the pyruvate content. In addition, the expression levels of two key fatty acid biosynthesis genes in the *HvTPS* RNAi specimens were higher than those in the controls, and the expression of one gene associated with lipid catabolism was decreased, as in the ds*TPS*‐injected larvae of *L. decemlineata* (Shi *et al*., 2016b). In short, various pathways reduce the amount of hemolymph monosaccharides in insect bodies. When one pathway is disrupted, another pathway may compensate for this disruption. For example, in this study, the biosynthesis of trehalose was inhibited by silencing *HvTPS*, resulting in the promotion of the synthesis of lipids in *H. vitessoides*.

In conclusion, we obtained a trehalose‐6‐phosphate synthase gene from *H. vitessoides*. Tissue‐ and stage‐specific expression analyses showed that *HvTPS* was highly expressed in the fat body and after pupation or before molting. Injection with a dose of 3.0 *μ*g of *dsHvTPS* was optimal with respect to RNAi efficiency in *H. vitessoides. HvTPS* silencing significantly inhibited *HvTPS* expression, and a sharp decline in the survival rate at the 5th instar larval–pupal stage as well as obviously abnormal or lethal phenotypes were observed. Furthermore, the silencing of *HvTPS* blocked the chitin biosynthesis pathway, but facilitated lipid biosynthesis. These results suggest that *HvTPS* plays a key role in metamorphosis as well as chitin and lipid biosynthesis in *H. vitessoides*.

## Disclosure

The authors declare no conflict of interest.

## Supporting information


**Figure S1**. Amino acid alignment of *trehalose‐6‐phosphate synthase* (TPS) sequences from *Spodoptera litura* (ADA63844), *Spodoptera exigua (*ABM66814), *Helicoverpa armigera* (XP_021201246), *Bombyx mori* (XP_004926812), *Amyelois transitella* (XP_013187220), *Gampsocleis gratiosa* (APZ77037), *Pogonomyrmex barbatus* (XP_011641245), *Linepithema humile* (XP_012234592), *Megachile Rotun‐data* (XP_003702415), *Apis cerana* (XP_016905400) and *Apis mellifera* (XP_00324923). Signature motifs unique to TPS (residues 175–179 and 405–410) are underlined.

**Table S1**. PCR primers used in this study.
**Table S2**. GenBank accession number in this study.
