# Supplementary material for: RNA interference of a trehalose‐6‐phosphate synthase gene reveals its roles in the biosynthesis of chitin and lipids in Heortia vitessoides (Lepidoptera: Crambidae)
Source: Insect Sci. 2018 Dec 11;27(2):212–23. doi: 10.1111/1744-7917.12650 (PMC7379938; doi:10.1111/1744-7917.12650)
Supplement: Supplementary file 1 — Figure S1. Amino acid alignment of trehalose‐6‐phosphate synthase (TPS) sequences from Spodoptera litura (ADA63844), Spodoptera exigua (ABM66814), Helicoverpa armigera (XP_021201246), Bombyx mori (XP_004926812), Amyelois transitella (XP_013187220), Gampsocleis gratiosa (APZ77037), Pogonomyrmex barbatus (XP_011641245), Linepithema humile (XP_012234592), Megachile Rotun‐data (XP_003702415), Apis cerana (XP_016905400) and Apis mellifera (XP_00324923). Signature motifs unique to TPS (residues 175–179 and 405–410) are underlined. [file INS-27-212-s001.doc]

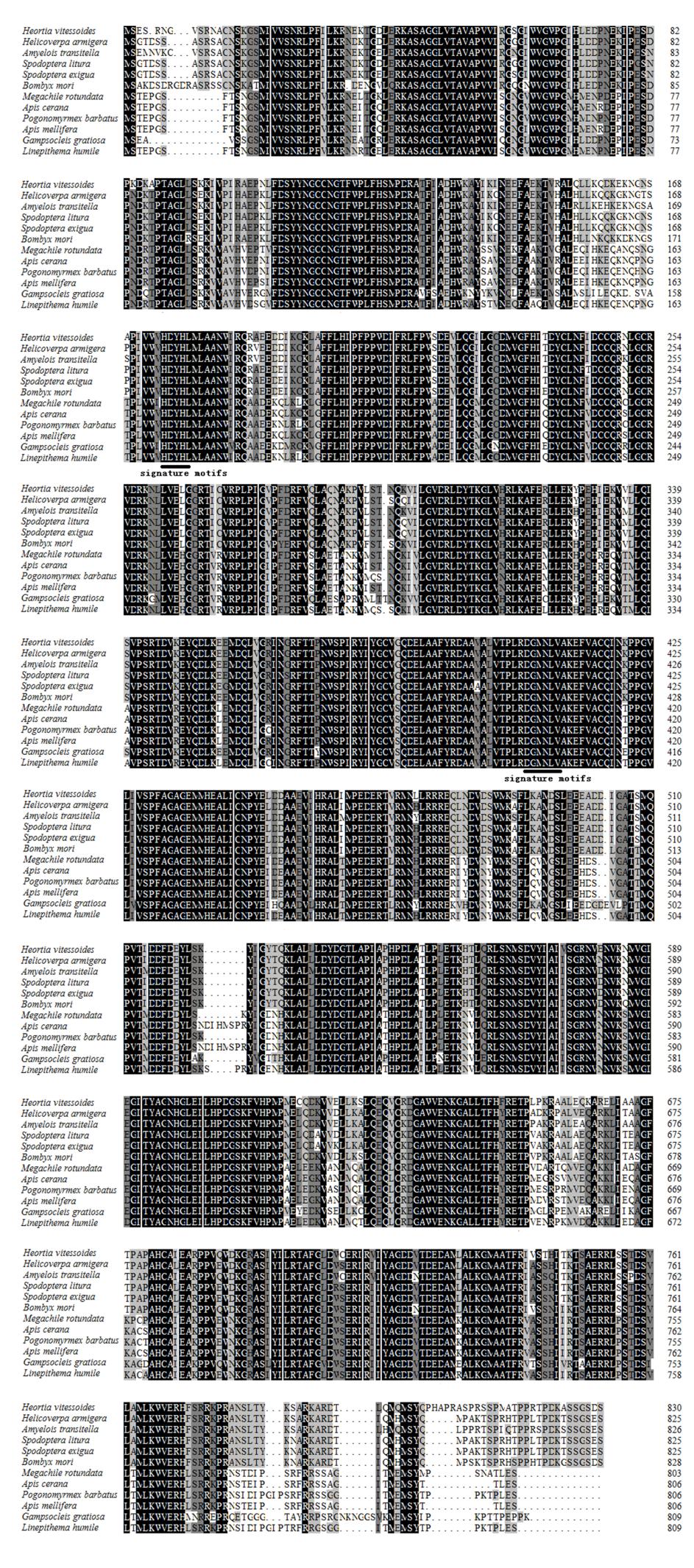


**Figure S1**. Amino acid alignment of *trehalose-6-phosphate synthase* (TPS) sequences from *Spodoptera litura* (ADA63844), *Spodoptera exigua (*ABM66814), *Helicoverpa armigera* (XP_021201246), *Bombyx mori* (XP_004926812), *Amyelois transitella* (XP_013187220), *Gampsocleis gratiosa* (APZ77037), *Pogonomyrmex barbatus* (XP_011641245), *Linepithema humile* (XP_012234592), *Megachile Rotun-data* (XP_003702415), *Apis cerana* (XP_016905400) and *Apis mellifera* (XP_00324923).Signature motifs unique to trehalose-6-phosphate synthase (TPS) (residues 175-179 and 405-410) are underlined.
