# Supplementary material for: RNA interference of a trehalose‐6‐phosphate synthase gene reveals its roles in the biosynthesis of chitin and lipids in Heortia vitessoides (Lepidoptera: Crambidae)
Source: Insect Sci. 2018 Dec 11;27(2):212–23. doi: 10.1111/1744-7917.12650 (PMC7379938; doi:10.1111/1744-7917.12650)
Supplement: Supplementary file 2 — Table S1. PCR primers used in this study. Table S2. GenBank accession number in this study. [file INS-27-212-s002.doc]

Table S1. PCR primers used in this study.

| Primers | Primer sequence |
| --- | --- |
| For real-time PCR |  |
| Q*HvTPS*-F | 5′-CGCTCCGAGATGGAATGAAT-3′ |
| Q*HvTPS*-R | 5′-GCTTCGTGCATCATCTCACC-3′ |
| Q*HvTre-1*-F | 5′-CAAGCCGGTCTACTGCGATA-3′ |
| Q*HvTre-1*-R | 5′-TCGTCGAGGATCTCTTGTGC-3′ |
| Q*HvTre-2*-F | 5′-AAGCAGAAGACCGCCATGTT-3′ |
| Q*HvTre-2*-R | 5′-GACATGACGACACCGTTGGT-3′ |
| Q*HvG6PI*-F | 5′-GGTGCACTTCGTCTCCAACA-3′ |
| Q*HvG6PI*-R | 5′-TAATGGTCTCCTGCGTGGTG-3′ |
| Q*HvUAP*-F | 5′-CTCATCTGCGCCTGATGGTA-3′ |
| Q*HvUAP*-R | 5′-TCAACAGAGTGAGCGTGCAA-3′ |
| Q*HvCHS-1*-F | 5′-GGGCACTTCTCCGAAAACT-3′ |
| Q*HvCHS-1*-R | 5′-CAACCCGACGAGCACATT-3′ |
| Q*HvCHS-2*-F | 5′-AACCTTGGTGCTGCTTGTG-3′ |
| Q*HvCHS-2*-R | 5′-TGCTCTGTCGCCTTTTGC-3′ |
| Q*HvACC*-F | 5′-GGCTTTGGTCGTGTTGGT-3′ |
| Q*HvACC*-R | 5′-TGTCGTCCTTGTTGCGGT-3′ |
| Q*HvFAS*-F | 5′-GTCGCCAAGCCAAAAGTC-3′ |
| Q*HvFAS*-R | 5′-TTGATGCCGAGCACGTT-3′ |
| Q*HvLIP1*-F | 5′-CCTCGTTGACCTGGAAGAA-3′ |
| Q*HvLIP1*-R | 5′-CCATTGACGGCAGTGTTG-3′ |
| *α-tubulin*-F | 5′-GATGCCCACAGACAAGACC-3′ |
| *α-tubulin*-R | 5′-AGTGTGGGTGGTCAGGATG-3′ |
| For cDNA cloning |  |
| 3-*HvTPS*-1 | 5′-AATGCCCGAGGATGAGAG-3′ |
| 3-*HvTPS*-2 | 5′-TTCCACTTCAGGGAGACGC-3′ |
| UPM | 5′-CTAATACGACTCACTATAGGGCAAGCAGTGGTATCAACGCAGAGT-3′ |
| NUP | 5′-AAGCAGTGGTATCAACGCAGAGT-3′ |
| For dsRNA synthesis |  |
| ds*HvTPS*-F | 5′-TAATACGACTCACTATAGGGCAACAGCAAAGGCAGTATG -3′ |
| ds*HvTPS*-R | 5′-TAATACGACTCACTATAGGGTTCTTCCTATCCACACGGC-3′ |
| *GFP-*F | 5′-TAATACGACTCACTATAGGGCAGTTCTTGTTGAATTAGATG-3′ |
| *GFP-*R | 5′-TAATACGACTCACTATAGGGTTTGGTTTGTCTCCCATGATG-3′ |

F: forward primer; R: reverse primer.

Table S2. GenBank accession number in this study.

| Gene | GenBank accession number |
| --- | --- |
| *HvTPS* | MG787167 |
| *HvTre-1* | MH050739 |
| *HvTre-2* | MG787168 |
| *HvG6PI* | MH050741 |
| *HvUAP* | MH050740 |
| *HvCHS-1* | MH142084 |
| *HvCHS-2* | MH142083 |
| *HvACC* | MH246984 |
| *HvFAS* | MH246983 |
| *HvLIP1* | MH246982 |
| *α-tubulin* | MG132200 |
